# *N*-(Pyrazin-2-yl)-4-toluidine

**DOI:** 10.1107/S160053680803729X

**Published:** 2008-11-26

**Authors:** Wan Ainna Mardhiah Wan Saffiee, Azila Idris, Zaharah Aiyub, Zanariah Abdullah, Seik Weng Ng

**Affiliations:** a Department of Chemistry, University of Malaya, 50603 Kuala Lumpur, Malaysia

## Abstract

The two aromatic systems in the title compound, C_11_H_11_N_3_, are inclined by 19.1 (1)°, whilst the angle at the central amino N atom is 130.3 (2)°. The amino group forms a hydrogen bond to the pyrazine N-4 atom of an adjacent mol­ecule, forming a chain motif.

## Related literature

For the structure of amino­pyrazine, see: Chao *et al.* (1976 ▶) and for that of *N*-(pyrazin-2-yl)-2-nitro­aniline; see: Parsons *et al.* (2006 ▶). For two monoclinic modifications of *N*-(pyrazin-2-yl)aniline, see: Abdullah & Ng (2008 ▶); Wan Saffiee *et al.* (2008 ▶).
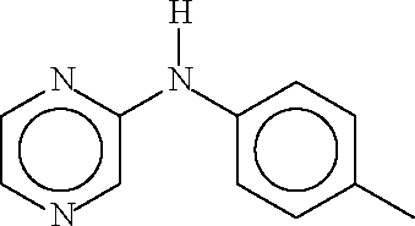


## Experimental

### 

#### Crystal data

C_11_H_11_N_3_
*M* *_r_* = 185.23
Monoclinic, 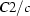

*a* = 21.7179 (7) Å
*b* = 7.5323 (3) Å
*c* = 12.0073 (5) Å
β = 105.790 (3)°
*V* = 1890.1 (1) Å^3^
*Z* = 8
Mo *K*α radiation
μ = 0.08 mm^−1^
*T* = 100 (2) K
0.30 × 0.20 × 0.05 mm

#### Data collection

Bruker SMART APEX diffractometer
Absorption correction: none
6057 measured reflections
2165 independent reflections
1437 reflections with *I* > 2σ(*I*)
*R*_int_ = 0.041

#### Refinement

*R*[*F* ^2^ > 2σ(*F* ^2^)] = 0.046
*wR*(*F* ^2^) = 0.135
*S* = 1.03
2165 reflections
132 parameters
H atoms treated by a mixture of independent and constrained refinement
Δρ_max_ = 0.27 e Å^−3^
Δρ_min_ = −0.26 e Å^−3^

### 

Data collection: *APEX2* (Bruker, 2007 ▶); cell refinement: *SAINT* (Bruker, 2007 ▶); data reduction: *SAINT*; program(s) used to solve structure: *SHELXS97* (Sheldrick, 2008 ▶); program(s) used to refine structure: *SHELXL97* (Sheldrick, 2008 ▶); molecular graphics: *X-SEED* (Barbour, 2001 ▶); software used to prepare material for publication: *publCIF* (Westrip, 2008 ▶).

## Supplementary Material

Crystal structure: contains datablocks global, I. DOI: 10.1107/S160053680803729X/sg2277sup1.cif

Structure factors: contains datablocks I. DOI: 10.1107/S160053680803729X/sg2277Isup2.hkl

Additional supplementary materials:  crystallographic information; 3D view; checkCIF report

## Figures and Tables

**Table 1:** Hydrogen-bond geometry (Å, °)

*D*—H⋯*A*	*D*—H	H⋯*A*	*D*⋯*A*	*D*—H⋯*A*
N1—H1⋯N3^i^	0.89 (2)	2.10 (2)	2.963 (2)	163 (2)

Symmetry code: (i) 
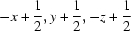
.

**Fig. 1.**
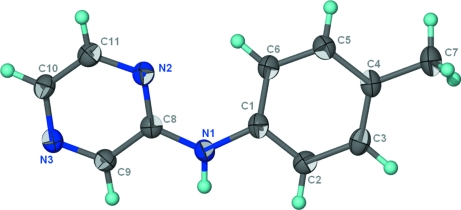
Thermal ellipsoid plot (Barbour, 2001) of C11H11N3 at the 70% probability level; hydrogen atoms are drawn as spheres of arbitrary radius.

## supplementary crystallographic information

### Experimental

Chloropyrazine (1 ml, 1.1 mmol) and 4-toluidine (1.2 g, 1.1 mmol) were heated at 423–433 K for 3 h. The solid was dissolved in water. The compound was extracted with ether. The ether extract was dried over sodium sulfate; evaporation of the solvent gave colorless crystals among some unidentified dark brown materials.

### Refinement

Carbon-bound H-atoms were placed in calculated positions (C—H 0.95–0.98 Å) and were included in the refinement in the riding model approximation, with *U*(H) fixed at 1.2–1.5*U*(C). The amino H-atom was located in a difference Fourier map, and was refined with a distance restraint of N–H 0.88±0.01 Å.

### Crystal data

**Table:**

C_11_H_11_N_3_	*F*_000_ = 784
*M**_r_* = 185.23	*D*_x_ = 1.302 Mg m^−^^3^
Monoclinic, *C*2/*c*	Mo *K*α radiation λ = 0.71073 Å
Hall symbol: -C 2yc	Cell parameters from 1368 reflections
*a* = 21.7179 (7) Å	θ = 2.9–27.2º
*b* = 7.5323 (3) Å	µ = 0.08 mm^−^^1^
*c* = 12.0073 (5) Å	*T* = 100 (2) K
β = 105.790 (3)º	Prism, colorless
*V* = 1890.1 (1) Å^3^	0.30 × 0.20 × 0.05 mm
*Z* = 8	

### Data collection

**Table:**

Bruker SMART APEX diffractometer	1437 reflections with *I* > 2σ(*I*)
Radiation source: fine-focus sealed tube	*R*_int_ = 0.041
Monochromator: graphite	θ_max_ = 27.5º
*T* = 100(2) K	θ_min_ = 2.0º
ω scans	*h* = −28→28
Absorption correction: None	*k* = −9→9
6057 measured reflections	*l* = −15→15
2165 independent reflections	

### Refinement

**Table:**

Refinement on *F*^2^	Secondary atom site location: difference Fourier map
Least-squares matrix: full	Hydrogen site location: inferred from neighbouring sites
*R*[*F*^2^ > 2σ(*F*^2^)] = 0.046	H atoms treated by a mixture of independent and constrained refinement
*wR*(*F*^2^) = 0.135	*w* = 1/[σ^2^(*F*_o_^2^) + (0.0652*P*)^2^ + 0.9346*P*] where *P* = (*F*_o_^2^ + 2*F*_c_^2^)/3
*S* = 1.03	(Δ/σ)_max_ = 0.001
2165 reflections	Δρ_max_ = 0.27 e Å^−^^3^
132 parameters	Δρ_min_ = −0.26 e Å^−^^3^
Primary atom site location: structure-invariant direct methods	Extinction correction: none

### Fractional atomic coordinates and isotropic or equivalent isotropic displacement parameters (Å^2^)

**Table:**

	*x*	*y*	*z*	*U*_iso_*/*U*_eq_	
N1	0.31486 (7)	0.7286 (2)	0.14663 (14)	0.0223 (4)	
H1	0.2746 (10)	0.754 (3)	0.1433 (17)	0.025 (5)*	
N2	0.39338 (7)	0.5068 (2)	0.20972 (13)	0.0215 (4)	
N3	0.30867 (7)	0.3328 (2)	0.31234 (14)	0.0240 (4)	
C1	0.34916 (8)	0.8610 (2)	0.10751 (15)	0.0209 (4)	
C2	0.31332 (8)	0.9994 (2)	0.04370 (15)	0.0225 (4)	
H2	0.2680	0.9977	0.0274	0.027*	
C3	0.34299 (9)	1.1385 (2)	0.00420 (15)	0.0243 (4)	
H3	0.3176	1.2305	−0.0396	0.029*	
C4	0.40936 (9)	1.1472 (2)	0.02707 (15)	0.0231 (4)	
C5	0.44431 (8)	1.0095 (2)	0.09091 (15)	0.0226 (4)	
H5	0.4897	1.0127	0.1081	0.027*	
C6	0.41565 (8)	0.8672 (2)	0.13069 (15)	0.0222 (4)	
H6	0.4412	0.7745	0.1734	0.027*	
C7	0.44137 (10)	1.3010 (3)	−0.01539 (18)	0.0302 (5)	
H7A	0.4876	1.2958	0.0203	0.045*	
H7B	0.4244	1.4129	0.0056	0.045*	
H7C	0.4328	1.2941	−0.0997	0.045*	
C8	0.33543 (8)	0.5719 (2)	0.20318 (15)	0.0201 (4)	
C9	0.29286 (8)	0.4818 (2)	0.25360 (16)	0.0231 (4)	
H9	0.2514	0.5298	0.2450	0.028*	
C10	0.36764 (8)	0.2678 (2)	0.32060 (16)	0.0231 (4)	
H10	0.3813	0.1616	0.3628	0.028*	
C11	0.40806 (9)	0.3538 (2)	0.26845 (16)	0.0235 (4)	
H11	0.4487	0.3022	0.2742	0.028*	

### Atomic displacement parameters (Å^2^)

**Table:**

	*U*^11^	*U*^22^	*U*^33^	*U*^12^	*U*^13^	*U*^23^
N1	0.0217 (8)	0.0194 (8)	0.0284 (9)	0.0007 (6)	0.0110 (7)	0.0031 (7)
N2	0.0249 (8)	0.0191 (8)	0.0223 (8)	0.0013 (6)	0.0095 (6)	−0.0011 (6)
N3	0.0254 (8)	0.0213 (8)	0.0262 (9)	−0.0016 (6)	0.0087 (7)	0.0019 (7)
C1	0.0275 (9)	0.0177 (9)	0.0191 (9)	−0.0009 (7)	0.0089 (7)	−0.0013 (7)
C2	0.0240 (9)	0.0224 (10)	0.0214 (10)	0.0019 (8)	0.0067 (7)	−0.0018 (8)
C3	0.0344 (10)	0.0191 (9)	0.0194 (10)	0.0024 (8)	0.0072 (8)	0.0004 (7)
C4	0.0316 (10)	0.0193 (9)	0.0200 (9)	−0.0032 (8)	0.0096 (8)	−0.0008 (7)
C5	0.0252 (9)	0.0222 (10)	0.0221 (10)	−0.0018 (8)	0.0093 (8)	−0.0018 (8)
C6	0.0261 (9)	0.0207 (9)	0.0204 (9)	0.0004 (7)	0.0071 (7)	0.0004 (7)
C7	0.0390 (11)	0.0250 (11)	0.0287 (11)	−0.0033 (9)	0.0128 (9)	0.0045 (8)
C8	0.0237 (9)	0.0196 (9)	0.0177 (9)	−0.0013 (7)	0.0065 (7)	−0.0023 (7)
C9	0.0222 (9)	0.0211 (9)	0.0267 (10)	0.0003 (7)	0.0079 (8)	0.0002 (8)
C10	0.0275 (9)	0.0187 (9)	0.0237 (10)	0.0022 (7)	0.0077 (8)	0.0003 (8)
C11	0.0264 (9)	0.0211 (10)	0.0238 (10)	0.0020 (8)	0.0080 (8)	−0.0020 (8)

### Geometric parameters (Å, °)

**Table:**

N1—C8	1.374 (2)	C4—C5	1.386 (3)
N1—C1	1.401 (2)	C4—C7	1.509 (3)
N1—H1	0.89 (2)	C5—C6	1.388 (2)
N2—C8	1.333 (2)	C5—H5	0.9500
N2—C11	1.344 (2)	C6—H6	0.9500
N3—C9	1.320 (2)	C7—H7A	0.9800
N3—C10	1.349 (2)	C7—H7B	0.9800
C1—C6	1.395 (2)	C7—H7C	0.9800
C1—C2	1.398 (2)	C8—C9	1.410 (2)
C2—C3	1.380 (3)	C9—H9	0.9500
C2—H2	0.9500	C10—C11	1.372 (3)
C3—C4	1.393 (3)	C10—H10	0.9500
C3—H3	0.9500	C11—H11	0.9500
			
C8—N1—C1	130.28 (15)	C5—C6—H6	120.2
C8—N1—H1	113.3 (13)	C1—C6—H6	120.2
C1—N1—H1	115.9 (14)	C4—C7—H7A	109.5
C8—N2—C11	115.60 (15)	C4—C7—H7B	109.5
C9—N3—C10	116.84 (15)	H7A—C7—H7B	109.5
C6—C1—C2	118.44 (16)	C4—C7—H7C	109.5
C6—C1—N1	124.95 (16)	H7A—C7—H7C	109.5
C2—C1—N1	116.58 (15)	H7B—C7—H7C	109.5
C3—C2—C1	120.81 (16)	N2—C8—N1	121.33 (16)
C3—C2—H2	119.6	N2—C8—C9	121.10 (16)
C1—C2—H2	119.6	N1—C8—C9	117.57 (15)
C2—C3—C4	121.50 (17)	N3—C9—C8	122.23 (16)
C2—C3—H3	119.3	N3—C9—H9	118.9
C4—C3—H3	119.3	C8—C9—H9	118.9
C5—C4—C3	117.08 (16)	N3—C10—C11	120.51 (17)
C5—C4—C7	121.84 (16)	N3—C10—H10	119.7
C3—C4—C7	121.08 (17)	C11—C10—H10	119.7
C4—C5—C6	122.59 (16)	N2—C11—C10	123.68 (17)
C4—C5—H5	118.7	N2—C11—H11	118.2
C6—C5—H5	118.7	C10—C11—H11	118.2
C5—C6—C1	119.58 (17)		
			
C8—N1—C1—C6	−7.4 (3)	N1—C1—C6—C5	−177.39 (17)
C8—N1—C1—C2	174.81 (17)	C11—N2—C8—N1	179.29 (16)
C6—C1—C2—C3	0.2 (3)	C11—N2—C8—C9	−1.1 (2)
N1—C1—C2—C3	178.22 (16)	C1—N1—C8—N2	−13.4 (3)
C1—C2—C3—C4	−0.6 (3)	C1—N1—C8—C9	166.96 (17)
C2—C3—C4—C5	0.2 (3)	C10—N3—C9—C8	−1.1 (3)
C2—C3—C4—C7	−179.30 (17)	N2—C8—C9—N3	2.1 (3)
C3—C4—C5—C6	0.4 (3)	N1—C8—C9—N3	−178.20 (16)
C7—C4—C5—C6	179.96 (17)	C9—N3—C10—C11	−0.8 (3)
C4—C5—C6—C1	−0.7 (3)	C8—N2—C11—C10	−0.8 (3)
C2—C1—C6—C5	0.4 (3)	N3—C10—C11—N2	1.8 (3)

### Hydrogen-bond geometry (Å, °)

**Table:**

*D*—H···*A*	*D*—H	H···*A*	*D*···*A*	*D*—H···*A*
N1—H1···N3^i^	0.89 (2)	2.10 (2)	2.963 (2)	163 (2)

Symmetry codes: (i) −*x*+1/2, *y*+1/2, −*z*+1/2.

## References

[bb1] Abdullah, Z. & Ng, S. W. (2008). *Acta Cryst.* E**64**, o2106. 10.1107/S1600536808031954 PMC2959673 21580970

[bb2] Barbour, L. J. (2001). *J. Supramol. Chem.* **1**, 189–191.

[bb3] Bruker (2007). *APEX2* and *SAINT* Bruker AXS Inc., Madison, Wisconsin, USA.

[bb4] Chao, M., Schempp, E. & Rosenstein, R. D. (1976). *Acta Cryst.* B**32**, 288–290.

[bb5] Parsons, S., Wharton, S., McNab, H., Parkin, A. & Johnstone, R. (2006). Private communcation (refcode SEMSAF 610410). CCDC, Cambridge, England.

[bb6] Sheldrick, G. M. (2008). *Acta Cryst.* A**64**, 112–122. 10.1107/S0108767307043930 18156677

[bb7] Wan Saffiee, W. A. M., Idris, A., Abdullah, Z., Aiyub, Z. & Ng, S. W. (2008). *Acta Cryst.* E**64**, o2105. 10.1107/S1600536808031942 PMC2959679 21580969

[bb8] Westrip, S. P. (2008). *publCIF* In preparation.

